# Moving Object Detection under a Moving Camera via Background Orientation Reconstruction

**DOI:** 10.3390/s20113103

**Published:** 2020-05-30

**Authors:** Wenlong Zhang, Xiaoliang Sun, Qifeng Yu

**Affiliations:** College of Aerospace Science and Engineering, National University of Defense Technology, Changsha 410073, China; wenlong@nudt.edu.cn (W.Z.); yuqifeng@nudt.edu.cn (Q.Y.)

**Keywords:** moving object detection, orientation field, background reconstruction, Poisson fusion, motion saliency

## Abstract

Moving object detection under a moving camera is a challenging question, especially in a complex background. This paper proposes a background orientation field reconstruction method based on Poisson fusion for detecting moving objects under a moving camera. As enlightening by the optical flow orientation of a background is not dependent on the scene depth, this paper reconstructs the background orientation through Poisson fusion based on the modified gradient. Then, the motion saliency map is calculated by the difference between the original and the reconstructed orientation field. Based on the similarity in appearance and motion, the paper also proposes a weighted accumulation enhancement method. It can highlight the motion saliency of the moving objects and improve the consistency within the object and background region simultaneously. Furthermore, the proposed method incorporates the motion continuity to reject the false positives. The experimental results obtained by employing publicly available datasets indicate that the proposed method can achieve excellent performance compared with current state-of-the-art methods.

## 1. Introduction

Moving object detection is incorporated in numerous applications, such as monitoring systems, unmanned aerial vehicles (UAVs), and automatic pilots [1]. The accurate position and shape information of a moving object are of great significance for subsequent tracking and recognition. Based on the camera platform being mobile or immobile, moving object detection can be classified into two categories: static camera and moving camera. Detecting moving objects under a static camera has been well studied. Background modelling methods [2,3,4] can achieve excellent performance in detecting moving object detection under a static camera. Thus, some methods [5,6,7,8] used motion compensation to adapt the background modeling to detect moving object under a moving camera. By assuming the background can be approximated by one or more dominant planes, the motion compensation methods usually use 2D parametric transformation [9,10] to register the current image with the background image. To eliminate the influence of the feature points on the object, [6] proposed a two-layer optimization method to estimate the affine transformation model. The methods [7,8,11] used two models for background and object to reduce the errors introduced in the motion compensation. Kim [12] used a spatial–temporal distributed Gaussian model to eliminate the false positives by registration error and background adaptation problem. Because of the complex background, the motion compensation by a 2D parametric transformation may be invalid for a scene with great depth variation. Detecting moving objects under a moving camera is much more difficult than that under a static camera. However, detecting moving objects under an unconstrained camera with high accuracy and robustness is very helpful for applications such as UAVs and automatic pilots. Thus, this study focuses on detecting moving object under an unconstrained camera accurately and reliably. Although many different methods have been proposed, the accuracy in detecting moving objects under an unconstrained camera is still unsatisfactory, especially for moving objects in a complex background. Usually, the moving object is detected based on the motion difference with the background, where the motion of the background and object are estimated by calculating the optical flow between adjacent frames. Therefore, the difference in optical flow is used to distinguish the object from the background. The motion projected on the image of the background depends on the distance to the camera, i.e., the depth of the scene. The magnitude of the optical flow for the pixels in the background region may be different, although they share the same real-world motion. The orientation of the optical flow is not dependent on the depth of the scene [13]. The variation of optical flow orientation in the background region is continuous in the spatial domain. According to this characteristic of background optical flow, this paper detects the moving object in the orientation field.

This paper proposes a background orientation reconstruction method to detect moving objects in the orientation field. The proposed method modifies the gradient of the original orientation field by removing the object orientation. Then, the background orientation field is reconstructed through Poisson fusion [14]. According to the difference between the original and reconstructed orientation field, this paper calculates the motion saliency map. For detecting all moving objects completely, the motion saliency map is enhanced through spatially weighted accumulation. Fundamentally, pixels belonging to the same category (moving object or background) are similar in appearance or motion in the corresponding neighborhood, and the weight can be defined for measuring the similarities. Based on this fundamental observation, this study proposes a spatially weighted accumulation method for enhancing the difference between the moving object and the background. Furthermore, this study incorporates the continuity of an object’s motion in the temporal domain for rejecting false positives.

## 2. Related Works

The conventional methods for moving object detection under a moving camera are generally based on the motion difference of the moving object and the background. The motion of the moving object comprises its own motion and the camera platform’s motion, while the motion of the background is solely caused by the camera platform. The motion cues are generally obtained based on the optical flow estimation between the adjacent frames.

Yazdi et al. [15] classified the methods into four categories: background subtraction, trajectory classification, low rank and sparse matrix decomposition, and object tracking. Chapel et al. [16] made a comprehensive review of methods for detecting moving objects under a moving camera, which were categorized into eight different approach groups. Depending on the density of the optical flow, this paper categorizes conventional methods for moving object detection under an unconstrained camera into two types: sparse optical flow methods [17,18,19,20,21,22,23] and dense optical flow methods [13,24,25,26,27,28,29,30,31,32,33,34].

The methods [17,23] clustered sparse sample pixels based on the optical flows and other spatial features as the moving object and background. Considering the classified pixels as seeds, they segmented the moving objects from the background. Nonaka et al. [21] used three distances to cluster the trajectories, and the cluster is classified according to the shape and size. To enhance the robustness of the extracted motion information, long trajectories have been used in some methods to detect the moving objects [18,19,20,22]. Sheikh et al. [18] calculated the basis vectors of the background trajectories based on the rank constraint to extract the trajectories of the moving objects. Spectral clustering was used to segment the moving object in [19,20] based on the affinities between the long trajectories. The spatial–temporal information was used to segment the moving objects densely based on the segmented sparse trajectories. However, the sparse optical flow methods can achieve satisfactory detection only if there are enough sampling pixels in the moving object region. This requirement can be easily met for large-sized moving objects but not for small-sized ones. Hence, such methods cannot easily detect small-sized moving objects.

Dense optical flow methods estimate the optical flow for each pixel between adjacent frames. The motion characteristics of the moving object and the background are subsequently extracted from the dense optical flow. Then, these methods calculate the motion saliency map to highlight the moving objects for detection. Gao et al. [24] measured the saliency by counting the histogram of the motion features. Huang et al. [25] adopted the homograph transformation to model the background’s motion based on the estimated optical flow. Based on the difference between the estimated optical flow and constructed optical flow by employing the homograph transformation, the motion saliency can be calculated for each pixel. Sajid et al. [26] reconstructed the background motion by a low-rank approximation. The probability of the moving object is estimated by the error between the reconstructed background motion and actual motion. The multi-view geometry constraint was used to distinguish between the object and background. Zhou et al. [27] detected moving targets under moving stereo cameras. The motion difference map, defined as the Residual Image Motion Flow, is calculated by the difference between the Measured Optical Flow and Global Image Motion Flow. The Global Image Motion Flow is obtained through geometric constraints of the moving camera. Finally, the motion likelihood, color, and depth cues are combined in the Markov Random Field (MRF) framework for moving object segmentation by graph-cut. Namdev et al. [28] used motion vectors from dense optical flow and motion potentials based on multi-view geometry to form a graph model. Then, a graph-based segmentation algorithm clustered nodes of similar potentials to create the eventual motion segments. The magnitude of the optical flow is dependent on the scene depth, whereas the orientation of the optical flow is independent of the scene depth. Narayana et al. [13] employed the orientation of the optical flow to calculate the motion saliency. Bideau et al. [29] estimated and eliminated the interferences caused by camera rotation for obtaining the orientation field of the optical flow, which is independent of the scene depth. In another study [30], the local spatial difference of the optical flow was used for calculating the saliency of the moving object’s contour, which was then employed for approximately selecting the object’s pixels. Chen et al. [31] proposed a context-aware motion descriptor based on the histogram of the orientation of the optical flow in a certain neighborhood, and the descriptor was employed for detecting the moving object’s contour. Wu et al. [32] constructed the dense particle trajectories based on the optical flow of multiple frames. Then, the motion saliency is calculated by comparing the trajectories with the extracted dominant motion components through the algorithm Reduced Singular Value Decomposition (RSVD) [34]. Zhu et al. [33] formulated the problem as a multi-label segmentation problem by modeling moving objects in different layers. An independent processing layer was assigned to each moving object and background.

To the best of our knowledge, the methods based on dense optical flow can achieve better performance in detecting and segmenting moving objects under an unconstrained camera. However, the two types of methods all have trouble in distinguishing the moving objects (relative the background) in a complex background. Hence, in this study, a background orientation reconstruction method is proposed for detecting the moving objects completely under a moving camera in a complex background. This paper also proposes an enhancement algorithm to highlight the motion saliency map for the moving objects and to smooth the region within the object and background simultaneously. Furthermore, the false positives are rejected based on the continuity of object motion in the temporal domain in this study.

## 3. Methodology

This paper proposes a moving object detection algorithm under a moving camera based on background orientation reconstruction. Firstly, the orientation of the optical flow between adjacent frames was calculated. Then, the original orientation field was modified in the gradient domain to remove the object region. Finally, the background orientation field was reconstructed based on the modified gradient through Poisson fusion [14]. The motion saliency can be obtained by the difference between the reconstructed background orientation and original orientation. Additionally, the motion saliency is enhanced through the spatially weighted accumulation of the neighborhood pixels. The detected result can be obtained by thresholding the enhanced motion saliency map. Furthermore, the continuity of the object’s motion in the temporal domain is incorporated for rejecting false detections.

### 3.1. Poisson Fusion

**Figure 1:**
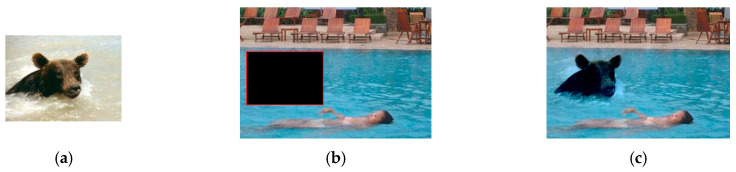
The sketch map of a Poisson fusion process: (**a**) is the source image; (**b**) is the fusion region Ω in the target image; and (**c**) is the desired image f fused into the target image $f^{*}$.

As shown in Figure 1, the Poisson fusion [14] solves the problem of seamlessly fusing the source image into the target image, meanwhile reserving the gradient information of the source image as far as possible.

To solve the problem, Pérez et al. [14] took the gradient field g of the source image as the guidance; the source image boundary ∂Ω of fusion region Ω served as a hard constraint for the desired image f. Then, the problem can be modeled as the following mathematical problem:

(1) $$\underset{f}{\min}\iint_{\Omega }{\left|\nabla f-g\right|}^{2}\;\;with\;\;f|{}_{\partial \Omega }=f^{*}|{}_{\partial \Omega }$$

where ∇f denotes the gradient field of the desired image f.

Solving the above problem, Equation (1) can obtain the following Poisson equation with Dirichlet boundary condition as follows:

(2) $$\Delta f|{}_{\Omega }=div\left(g\right)|{}_{\Omega }\;\;with\;\;f|{}_{\partial \Omega }=f^{*}|{}_{\partial \Omega }$$

where $div\left(g\right)=\frac{\partial g_{x}}{\partial x}+\frac{\partial g_{y}}{\partial y}$ is the divergence of the gradient field $g=\left(g_{x},g_{y}\right)$.

The variables in Equation (2) are discrete in the image domain. Equation (2) can be solved discretely by the five-point interpolation method in the numerical method of partial differential equations [35]. The calculation process is shown in the Appendix A, and the result of the Poisson fusion is shown in Figure 1c.

### 3.2. Motion Saliency through Background Orientation Reconstructed

This study uses the optical flow orientation to distinguish the background and moving object because it does not depend on the scene depth for the background region. The orientation field of the background varies continuously in the spatial domain. Thus, the value of the gradient of the background orientation field tends to be continuous. According to this property, the gradient field of the background orientation can be obtained by smoothing the mutant in the original gradient of the orientation field.

The angle of the optical flow between the adjacent frames is used to describe the orientation field. Then, the gradient of the orientation field can be obtained as follows:

(3) $$\begin{array}{l} g_{i,j}^{x}=\theta_{i,j+1}-\theta_{i,j}\\ g_{i,j}^{y}=\theta_{i+1,j}-\theta_{i,j} \end{array}$$

where θ represents the angle of the original optical flow orientation. $g_{i,j}^{x}$ and $g_{i,j}^{y}$ denote the gradient of the orientation field in the position (i,j) along the horizontal and vertical directions, respectively.

This study defines the mutant as the local maximum in the gradient of the orientation field. The smaller value of the gradient in the neighborhood is used to substitute the local maximum to eliminate the mutant. This paper eliminates the mutant of $g_{i,j}^{x}$ along the horizontal direction as follows:

(4) $${\hat{g}}_{i,j}^{x}=\left\{ \begin{array}{l} g_{i,j-1\;\;}^{x}\;\;\left|g_{i,j}^{x}\right|>\left|g_{i,j-1\;\;}^{x}\right|\;and\;\left|g_{i,j}^{x}\right|>\left|g_{i,j+1\;\;}^{x}\right|\;and\;\left|g_{i,j+1}^{x}\right|>\left|g_{i,j-1\;\;}^{x}\right|\\ g_{i,j+1\;\;}^{x}\;\;\left|g_{i,j}^{x}\right|>\left|g_{i,j-1\;\;}^{x}\right|\;and\;\left|g_{i,j}^{x}\right|>\left|g_{i,j+1\;\;}^{x}\right|\;and\;\left|g_{i,j+1}^{x}\right|<\left|g_{i,j-1\;\;}^{x}\right|\\ g_{i,j\;\;}^{x}\;\;otherwise \end{array}\right.$$

where ${\hat{g}}_{i,j}=\left({\hat{g}}_{i,j}^{x},{\hat{g}}_{i,j}^{y}\right)$ denotes the modified gradient in the position (i,j).

The vertical gradient field $g_{i,j}^{y}$ is conducted in a similar manner.

Now, the gradient field ${\hat{g}}_{i,j}=\left({\hat{g}}_{i,j}^{x},{\hat{g}}_{i,j}^{y}\right)$ with no mutant is obtained, which serves as the gradient of the background orientation angle.

Assuming that the pixels in the image boundaries are background, similar to Equation (1), the minimization problem can be set up with respect to the angle of the background orientation as follows:

(5) $$\underset{\theta^{b}}{\min}\iint_{\Omega }{\left|\nabla \theta^{b}-\hat{g}\right|}^{2}\;\;with\;\;\theta^{b}|{}_{\partial \Omega }=\theta |{}_{\partial \Omega }$$

where $\theta^{b}$ denotes the angle of the reconstructed background orientation; and ∂Ω and Ω represent the image boundaries and interior region, respectively.

The motion saliency $M_{i,j}$ is defined as the absolute value of the angle difference between the reconstructed background orientation angle and the original orientation angle.

(6) $$M_{i,j}=\left|\theta_{i,j}-\theta_{i,j}^{b}\right|$$

**Figure 2:**
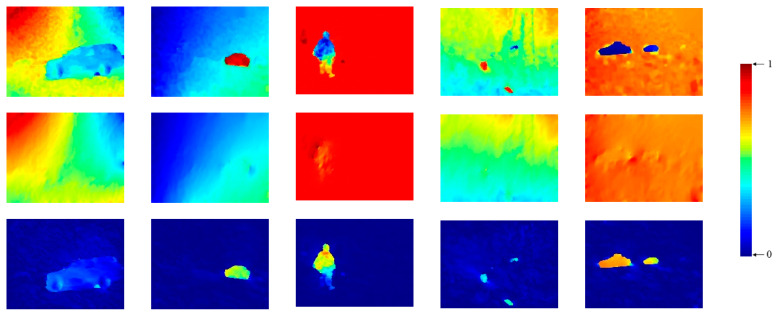
Some examples of reconstructed background orientation angle (**middle row**) and motion saliency map (**bottom row**). The maps in the (**top row**) are the original orientation angle.

As shown in Figure 2, the algorithm proposed in this section can reconstruct the orientation field of the background with relative accuracy (see the middle rows). The moving objects can be highlighted in the motion saliency map calculated by Equation (6).

### 3.3. Enhancement Algorithm Based on Weighted Spatial Accumulation

As shown in Figure 2 (the first and fourth columns), the moving object may be indistinguishable in that the motion saliency calculated by Equation (6) may be low. There also may be inconsistencies within the background and object region. This section’s objective is to enhance the motion saliency map for detecting moving objects as completely as possible. The motion saliency of a pixel can be enhanced by the accumulation of its surrounding pixels in the spatial domain. The increased amount for the moving object is much larger than the background, and the motion saliency within the object or background would become much more uniform. In order to find the pixels belonging to the same target for more exact accumulation, this study enhances the motion saliency map by weighted accumulation. The pixels that belong to the same object usually are similar in terms of appearance and motion. In this study, the weight of each pixel was determined by measuring the appearance and motion similarity between each pixel pair in a certain neighborhood.

Appearance similarity: The appearance similarity between a pixel $p_{\alpha }$ and its neighborhood pixel $p_{\beta }$ can be calculated by incorporating the color difference $S_{i,j}^{c}$, as follows:

(7) $$S_{\alpha ,\beta }^{c}=\exp\left(-\frac{{\left(R_{\alpha }-R_{\beta }\right)}^{2}+{\left(G_{\alpha }-G_{\beta }\right)}^{2}+{\left(B_{\alpha }-B_{\beta }\right)}^{2}}{\sigma_{1}}\right)\;\;\;\;\;\;\;\;\;p_{\beta }\in N_{\alpha }$$

where $\left(R_{\alpha },G_{\alpha },B_{\alpha }\right)$, respectively, denote the red, blue, and green value of $p_{\alpha }$; $N_{\alpha }$ represents the n×n neighborhoods of $p_{\alpha }$; $p_{\beta }$ denotes the neighborhood pixel of $p_{\alpha }$; and $\sigma_{1}$ is a positive constant parameter and set to 25 in this study.

Motion similarity: The motion similarity $S_{\alpha ,\beta }^{m}$ between $p_{\alpha }$ and $p_{\beta }$ can be determined by incorporating the optical flow difference, as follows:

(8) $$S_{\alpha ,\beta }^{m}=\exp\left(-\frac{{\left(u_{\alpha }-u_{\beta }\right)}^{2}+{\left(v_{\alpha }-v_{\beta }\right)}^{2}}{\sigma_{2}}\right)$$

where $\left(u_{\alpha },v_{\alpha }\right)$ and $\left(u_{\beta },v_{\beta }\right)$, respectively, denote the optical flow vector of $p_{\alpha }$ and $p_{\beta }$. Furthermore, $\sigma_{2}$ represents the variance of the Gaussian function, and its value is set to 5 in this study.

The similarity between $p_{\alpha }$ and $p_{\beta }$ can be obtained from the product of $S_{\alpha ,\beta }^{c}$ and $S_{\alpha ,\beta }^{m}$, as shown in Equation (9).

(9) $$S_{\alpha ,\beta }=S_{\alpha ,\beta }^{c}\times S_{\alpha ,\beta }^{m}$$

If the similarity is directly applied as the cumulative weight, the pixels near the object’s contour and those inside the object will be enhanced by different extents owing to varying numbers of object pixels in their corresponding neighborhoods. To avoid the problem of enhancement being dependent on the pixel position, the summation of the similarities of the neighborhood pixels is normalized to the number of the neighborhoods in this study. Thus, the accumulation of weight can be defined as

(10) $$W_{\alpha ,\beta }=\frac{\left(n\times n-1\right)\times {\hat{S}}_{\alpha ,\beta }}{\sum_{j}{\hat{S}}_{\alpha ,\beta }},\;\mathrm{where}\;{\hat{S}}_{\alpha ,\beta }=\frac{S_{\alpha ,\beta }-\underset{j}{\min}S_{\alpha ,\beta }}{\underset{\beta }{\max}S_{\alpha ,\beta }-\underset{\beta }{\min}S_{\alpha ,\beta }}.$$

where the value of n is set to 9 in this study.

The enhanced motion saliency ${\hat{M}}_{\alpha }$ of pixel $p_{\alpha }$ can be obtained through the spatial accumulation of its neighborhoods with the similarity weight, as shown in Equation (11).

(11) $${\hat{M}}_{\alpha }=M_{\alpha }+\sum_{p_{\beta }\in N_{\alpha },p_{\beta }\neq p_{\alpha }}W_{\alpha ,\beta }\times M_{\beta }$$

where $M_{\alpha }$ and $M_{\beta }$ donate the motion saliency of pixel $p_{\alpha }$ and $p_{\beta }$, respectively.

**Figure 3:**
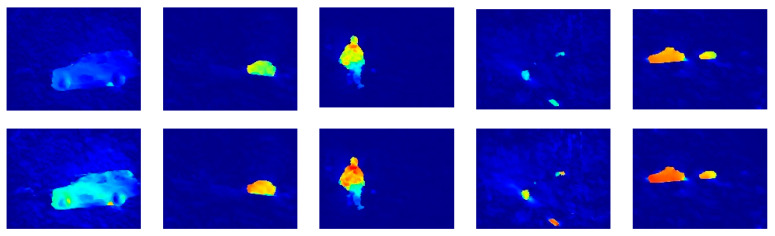
Motion saliency maps before (**top row**) and after (**bottom row**) enhancement (the color map is the same as in Figure 2).

As shown in Figure 3, the motion saliency of a moving object can be enhanced by Equation (11). To detect all moving objects as completely as possible, this paper adopts a relatively small threshold. The value of the threshold T is determined by incorporating the mean value *m* and standard deviation *σ* of the enhanced motion saliency, as follows:

(12) T=m+σ

### 3.4. False Positives Rejection Based on Motion Continuity in the Temporal Domain

False positives are usually caused by a cluttered background, inaccuracy in the estimated optical flow, and a flawed threshold algorithm, which may exist in the detected results. This study utilizes motion continuity in the temporal domain to address the issue. The trajectory of a real moving object is continuous in the temporal domain, but a false alarm does not have a continuous trajectory. In this study, false detection in the current frame is rejected according to the detection in the next frame, and the correspondence between the current frame and the next frame is established through the optical flow. If a detected region in the current frame is consistent with the next frame’s detection, it is determined as a true moving object; otherwise, it is determined as a false positive. Considering the variation of the moving object shape caused by changes in viewing and the object’s movement, the consistency between the adjacent frames is measured by the area overlap ratio. If the overlap ratio is greater than the threshold λ, the region is identified as a true moving object, and vice versa.

(13) $$O_{t,m}=\left\{ \begin{array}{l} 1\;\;\;\;\;\frac{\left|{\hat{O}}_{t+1,m}\cap O_{t+1}\right|}{\left|O_{t,m}\right|}>\lambda \\ 0\;\;\;\;\;\;\;otherwise \end{array}\right.$$

where $O_{t,m}$ represents the *m*th detected object region in the current frame, and ${\hat{O}}_{t+1,m}$ denotes the projected region in the next frame from the object $O_{t,m}$ based on the optical flow. Furthermore, $O_{t+1}$ represents the detected object region in the next frame, and |·| represents the number of total pixels within the region. In this study, the value of the threshold λ is set to 0.3.

**Figure 4:**
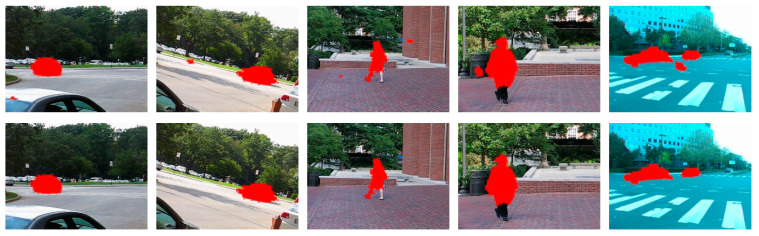
The detected results before (**top row**) and after (**bottom row**) false positives rejection (the red color denotes the detected objects).

As shown in Figure 4, there may exist some false positives in the background regions. The proposed false positives can effectively eliminate the false positives.

## 4. Experiment

To evaluate the performance of the proposed method, this study conducted experiments by employing the publicly available datasets moseg_dataset [20], BMS (Background Motion Subtraction) [32], and DAVIS (Densely Annotated Video Segmentation) [36]. The publicly available datasets comprise multiple image sequences, which contain moving objects in different complex backgrounds captured by moving cameras with different movements. The quantitative and qualitative comparisons were made with compared methods [5,25,29,30]. The codes of algorithms [5,25,29,30] were all downloaded from the authors’ homepage. The parameters suggested by the respective authors were incorporated into the compared methods. This paper calculated the optical flow in [25,29,30] and the proposed method by employing the same algorithm [37]. Furthermore, all the objects detected by the proposed algorithm were obtained by the same parameters, as described earlier.

### 4.1. Qualitative Comparison

**Figure 5:**
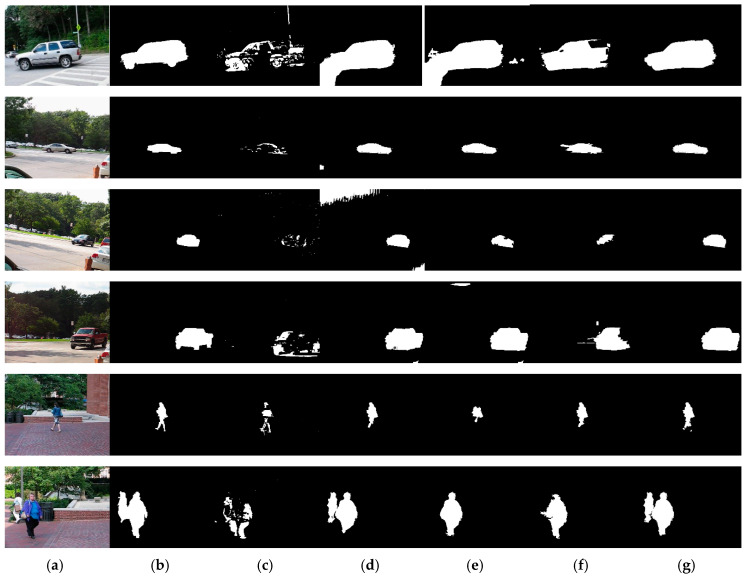
Samples of the detection results using the public dataset moseg_dataset [20]. (**a**) are the original images and (**b**) are the corresponding ground-truthed images; (**c**–**f**) are the results of methods in [5,25,29,30], respectively; and (**g**) images from the proposed method.

Figure 5 illustrates some detected results generated from different sequences of the dataset moseg_dataset [20]. As shown in Figure 5c, the detected objects by [5] lost the shape information, which can only be used to determine the approximate position of the objects. The detected results by [25] have some false positives in the background region because of the assumption that it is approximating the background by a plane. Some parts of the moving objects are missed in the results by [30], which loses the complete shape of the objects. The proposed method detects the moving objects much more completely and with less false positives.

**Figure 6:**
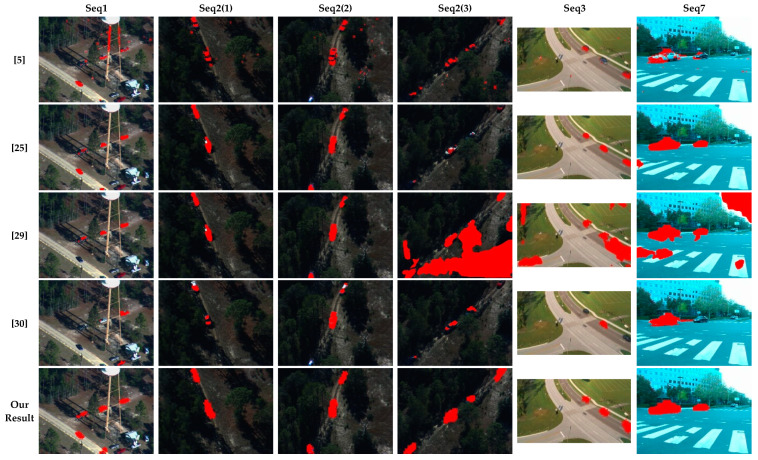
Some detected results on the public dataset BMS [32] of [5,25,29,30] and the proposed method.

Figure 6 illustrates some sample results on the dataset BMS [32] obtained by methods [5,25,29,30] and the proposed method. Seq2 was divided into three subsequences because the algorithm in [29,30] cannot process the entire sequence at once due to the memory constraint. As seen from Figure 6, the proposed method can detect the moving objects completely with less false positives.

**Figure 7:**
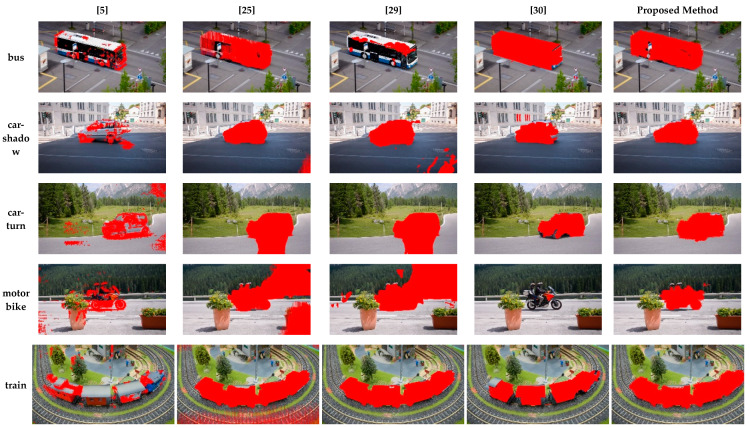
Some detected results from DAVIS [36] by the proposed and compared methods [5,25,29,30].

Some sample results on the dataset DAVIS [36] obtained by methods [5,25,29,30] and the proposed method are shown in Figure 7. As seen from Figure 7, the proposed method can detect the moving objects completely with less false positives. The detected results by [25] tend to have false positives in the background region. A similar problem appeared in the results by [29], such as car-shadow, car-turn, and motorbike. This problem does not occur in the detected result by the proposed method because of good adaptability to the change in scene depth.

### 4.2. Quantitative Comparison

In this study, the overlap rate was employed for quantitatively evaluating the accuracy of the detection algorithms. Given the detected object $D_{O}$ and the ground truth $G_{O}$, the overlap ratio γ is defined as

(14) $$\gamma =\frac{\left|D_{O}\cap G_{O}\right|}{\left|D_{O}\cup G_{O}\right|}$$

The obtained results are summarized in Table 1, wherein figures in boldface font represent the highest overlap rate, and figures in italic font represent the second-highest overlap rate. It can be observed from Table 1 that the proposed method achieves the best performance for all the datasets. The proposed method is comparable to the state-of-the-art algorithms in [25,29,30]. Comparing to the algorithm described in [25], the proposed background orientation reconstruction algorithm does not assume that the background is approximated by planes. Therefore, the proposed method is able to adapt to more complex scenarios. The algorithm [29] has poor robustness in that the moving object may not be detected for a long period of time, such as Seq1 and Cars2. However, the proposed algorithm is more robust because of good adaptability to the variation of scene depth. Thus, our algorithm usually obtained a relatively high score for the sequences difficult to detect, such as Seq1, Seq2(3), Cars4, Cars9, and motorbike. Furthermore, it should be noted that the algorithm in [30] employs many frames in the future to segment the moving object in the current frame. The algorithm in [29] also uses complex optimization to estimate the background orientation. The complexity of the algorithm in [29] is much higher than that of the proposed algorithm.

There is one case in which the proposed method cannot work: Because only the orientation of the optical flow is used to calculate the motion difference between the object and background, the object moving in the same direction as the camera cannot be detected by the proposed method. The amplitude of the optical flow can be used as Supplementary Information to improve the proposed method.

### 4.3. Computational Efficiency

The proposed background orientation reconstruction method can be very efficient due to the coefficient matrix only depending on the resolution ratio of the image; so, it only needs to be solved once for one sequence because of the same size of the images within the same sequence. However, it needs a large amount of memory. Thus, this paper solves the Poisson equation every time for each frame, and the motion saliency enhancement method is also efficient owing to its high parallelism; it takes about 8 ms for processing each frame at a resolution of 480 × 640 by employing an NVIDIA GTX 1080 from Asus, Chongqing, china. The procedure of false positives rejection takes very little time. Table 2 shows the average computation time per frame with a resolution of 480 × 640 measured by an Intel Core i5-6200U, 2.4GHz PC from Lenovo, Beijing, china. The method [5] was implemented in C++ and the other four by MATLAB. The time spent on optical flow computation was excluded, which is required for all methods except the one in [5]. The computational efficiency of the proposed method is superior to that of the algorithms in [29,30], and the computational efficiency of the proposed method can be improved greatly through adopting a more advanced sparse equation solving method.

## 5. Conclusions

This paper proposes a novel method for detecting moving objects under a moving camera. This paper reconstructs the background orientation field through Poisson fusion based on the modified gradient and the orientation in image boundaries. The motion saliency map can be obtained by the difference between the original and reconstructed background orientation field. Based on the appearance and motion similarity, the proposed method enhances the motion saliency map through weighted accumulation in the spatial domain. Furthermore, the proposed method incorporates motion continuity in the temporal domain for rejecting false positives. Experimental results based on the publicly available datasets indicate that the proposed method can achieve an excellent performance through qualitative and quantitative comparison.

Since our paper only uses the optical flow orientation, the object moving in the same direction as the camera cannot be detected. In this situation, the amplitude of the optical flow between the moving object and background must be different, or the object is static. In the next work, we will use the amplitude as a check and supplement the scheme, to verify if there are missed objects, and to detect them. On the other hand, the thresholding method for motion saliency mapping is simple, which influences the accuracy of the proposed method. We will look at getting a more precise object from the motion saliency map.

## Figures and Tables

**Table 1:** The overlap rate (%) obtained after applying the methods in [5,25,29,30] and the proposed method.

	Method	[5]	[25]	[29]	[30]	Proposed Method
Sequence						
Seq1		26.41	21.82	*35.08*	8.49	36.40
Seq2(1)		34.55	50.49	63.66	33.88	*54.95*
Seq2(2)		39.54	25.89	58.16	42.24	*49.43*
Seq2(3)		24.40	*33.27*	24.85	24.24	49.27
Seq3		22.46	58.50	37.84	19.98	*41.54*
Seq7		39.15	87.63	21.26	60.40	*66.70*
Cars1		27.04	64.24	59.24	*68.03*	74.12
Cars2		10.45	19.98	65.35	1.07	*48.42*
Cars4		19.94	45.27	17.29	37.22	62.32
Cars6		18.32	*88.04*	89.17	80.54	80.30
Cars7		22.70	64.80	91.24	42.27	*81.05*
Cars8		27.45	83.20	80.69	71.69	*81.41*
Cars9		12.51	31.20	40.73	*41.05*	44.82
People1		37.97	*75.64*	76.57	55.44	70.34
People2		36.73	69.05	77.46	*75.52*	73.31
bear		11.61	37.34	*79.76*	83.68	70.18
bus		39.19	81.42	79.19	70.90	*80.49*
car-shadow		29.17	79.03	30.88	69.79	*74.50*
car-turn		48.02	64.10	65.33	68.63	*67.05*
motorbike		36.49	*49.92*	42.30	33.53	59.91
train		16.47	*75.83*	90.22	72.70	75.03
Average		27.65	57.46	*58.39*	50.54	63.88

**Table 2:** Time consumption of the proposed and other methods.

Method	[5]	[25]	[29]	[30]	Proposed Method
Time (ms)	46.21	28.94	1075.35	6042.88	854.96

## Appendix A

Five-point interpolation method for solving the Poisson equation in reconstructing the background orientation.

The problem is to solve following optimizing formula:

(A1) $$\underset{\theta^{b}}{\min}\iint_{\Omega }{\left|\nabla \theta^{b}-\hat{g}\right|}^{2}\;\;with\;\;\theta^{b}|{}_{\partial \Omega }=\theta |{}_{\partial \Omega }$$

Solving the above problem, we can obtain the following Poisson equation with Dirichlet boundary conditions:

(A2) $$\Delta \theta^{b}|{}_{\Omega }=div\left(\hat{g}\right)|{}_{\Omega }\;\;with\;\;\theta^{b}|{}_{\partial \Omega }=\theta |{}_{\partial \Omega }$$

The discrete second-order differential of $\theta^{b}$ along the x and y direction is as follows:

(A3) $$\begin{array}{l} \left(\frac{\partial^{2}\theta_{i,j}^{b}}{\partial x^{2}}\right)=\frac{1}{h^{2}}\left(\theta_{i,j+1}^{b}+\theta_{i,j-1}^{b}-2\theta_{i,j}^{b}\right)+O\left(h^{2}\right)\\ \left(\frac{\partial^{2}\theta_{i,j}^{b}}{\partial y^{2}}\right)=\frac{1}{h^{2}}\left(\theta_{i+1,j}^{b}+\theta_{i-1,j}^{b}-2\theta_{i,j}^{b}\right)+O\left(h^{2}\right) \end{array}$$

Here, h denotes the step of the discretization, which is usually set to 1 in image process.

If we ignore the error term $O\left(h^{2}\right)$ and set the boundary size to 1, the discrete Poisson equation is as follows:

(A4) $$\theta_{i,j+1}^{b}+\theta_{i,j-1}^{b}+\theta_{i+1,j}^{b}+\theta_{i-1,j}^{b}-4\theta_{i,j}^{b}=div\left({\hat{g}}_{i,j}\right)\;\;\;\;\;2\leq i\leq r-1;2\leq j\leq c-1$$

Here, r and c respectively denote the height and width of the orientation field.

Vectorizing $\theta^{b}$ by column priority as

$${\vec{\theta }}^{T}=[\theta_{2,2}\;\theta_{3,2}\cdots \theta_{r-1,2}\;\theta_{2,3}\;\cdots \theta_{r-1,c-1}]$$

Now, the following equation respect to ${\vec{\theta }}^{T}$ can be obtained:

(A5) $$A_{\left[\left(r-2)(c-2\right)\right]\times \left[\left(r-2)(c-2\right)\right]}\cdot \vec{\theta }=b_{\left(r-2)(c-2\right)}$$

where the vector b is depended on the gradient field $\hat{g}$ and the orientation angle of the pixels at the boundaries; and A is a positive definite symmetric matrix of order (r−2)(c−2), as follows:

$$A=\left[\begin{array}{ccccc} B & I & & & \\ I & B & I & & \\ & \ddots & \ddots & \ddots & \\ & & I & B & I\\ & & & I & B \end{array}\right]$$

Here, I is unit matrix of order (r−2); and B is a symmetric matrix of order (r−2), as follows:

$$B_{\left(r-2)\times (r-2\right)}=\left[\begin{array}{ccccc} -4 & 1 & & & \\ 1 & -4 & 1 & & \\ & \ddots & \ddots & \ddots & \\ & & 1 & -4 & 1\\ & & & 1 & -4 \end{array}\right]$$

The coefficient matrix A in Equation (A5) is a large sparse matrix, depending only on the size of the image.

## References

[1] Royden C.S., Moore K.D. (2012). Use of speed cues in the detection of moving objects by moving observers. Vis. Res..

[2] Barnich O., Van Droogenbroeck M. (2010). ViBe: A universal background subtraction algorithm for video sequences. IEEE Trans. Image Process..

[3] Elgammal A., Harwood D., Davis L. (2000). Non-parametric model for background subtraction. Proceedings of the 6th European Conference on Computer Vision.

[4] Yong H., Meng D., Zuo W., Zhang K. (2018). Robust online matrix factorization for dynamic background subtraction. IEEE Trans. Pattern Anal. Mach. Intell..

[5] Yi K.M., Yun K., Kim S.W., Chang H.J., Choi J.Y., Jeong H. (2013). Detection of moving objects with non-stationary cameras in 5.8ms: Bringing motion detection to your mobile device. Computer Vision & Pattern Recognition Workshops.

[6] Wan Y., Wang X., Hu H. (2014). Automatic moving object segmentation for freely moving cameras. Math. Probl. Eng..

[7] Wu M., Peng X., Zhang Q. (2011). Segmenting moving objects from a freely moving camera with an effective segmentation cue. Meas. Sci. Technol..

[8] Kurnianggoro L., Yu Y., Hernandez D., Jo K.-H. (2016). Online Background-Subtraction with Motion Compensation for Freely Moving Camera. International Conference on Intelligent Computing.

[9] Odobez J.M., Bouthemy P. (1997). Separation of moving regions from background in an image sequence acquired with a mobile camera. Video Data Compression for Multimedia Computing: Statistically Based and Biologically Inspired Techniques.

[10] Hartley R., Zisserman A. (2003). Multiple View Geometry in Computer Vision.

[11] Zhang X., Sain A., Qu Y., Ge Y., Hu H. (2019). Background subtraction based on integration of alternative cues in freely moving camera. IEEE Trans. Circuits Syst. Video Technol..

[12] Kim S.W., Yun K., Yi K.M., Kim S.J., Choi J.Y. (2012). Detection of moving objects with a moving camera using non-panoramic background model. Mach. Vis. Appl..

[13] Narayana M., Hanson A., Learned-Miller E. Coherent motion segmentation in moving camera videos using optical flow orientations. Proceedings of the 2013 IEEE International Conference on Computer Vision.

[14] Pérez P., Gangnet M., Blake A. (2003). Poisson image editing. ACM Trans. Graphics.

[15] Yazdi M., Bouwmans T. (2018). New trends on moving object detection in video images captured by a moving camera: A survey. Comput. Sci. Rev..

[16] Chapel M.-N., Bouwmans T. (2020). Moving objects detection with a moving camera: A comprehensive review. arXiv.

[17] Kim J., Wang X., Wang H., Zhu C., Kim D. (2012). Fast moving object detection with non-stationary background. Multimedia Tools Appl..

[18] Sheikh Y., Javed O., Kanade T. Background subtraction for freely moving cameras. Proceedings of the 2009 IEEE 12th International Conference on Computer Vision.

[19] Brox T., Malik J. (2010). Object segmentation by long term analysis of point trajectories. Proceedings of the 11th European Conference on Computer Vision.

[20] Ochs P., Malik J., Brox T. (2013). Segmentation of moving objects by long term video analysis. IEEE Trans. Pattern Anal. Mach. Intell..

[21] Nonaka Y., Shimada A., Nagahara H., Taniguchi R.-I. Real-time foreground segmentation from moving camera based on case-based trajectory classification. Proceedings of the 2013 2nd IAPR Asian Conference on Pattern Recognition.

[22] Elqursh A., Elgammal A. (2012). Online moving camera background subtraction. Appl. Evol. Comput..

[23] Bugeau A., Perez P. (2009). Detection and segmentation of moving objects in complex scenes. Comput. Vis. Image Underst..

[24] Gao Z., Tang W., He L. (2016). Moving object detection with moving camera based on motion saliency. J. Comput. Appl..

[25] Huang J., Zou W., Zhu J., Zhu Z. (2018). Optical flow based real-time moving object detection in unconstrained scenes. arXiv.

[26] Sajid H., Cheung S.-C.S., Jacobs N. (2019). Motion and appearance based background subtraction for freely moving cameras. Signal. Process. Image Commun..

[27] Zhou D., Frémont V., Quost B., Dai Y., Li H. (2017). Moving object detection and segmentation in urban environments from a moving platform. Image Vis. Comput..

[28] Namdev R.K., Kundu A., Krishna K.M., Jawahar C.V. Motion segmentation of multiple objects from a freely moving monocular camera. Proceedings of the 2012 IEEE International Conference on Robotics and Automation.

[29] Bideau P., Learned-Miller E. It’s Moving! A probabilistic model for causal motion segmentation in moving camera videos. Proceedings of the 14th European Conference on Computer Vision.

[30] Papazoglou A., Ferrari V. Fast object segmentation in unconstrained video. Proceedings of the 2013 IEEE International Conference on Computer Vision.

[31] Chen T., Lu S. (2016). Object-level motion detection from moving cameras. IEEE Trans. Circuits Syst. Video Technol..

[32] Wu Y., He X., Nguyen T.Q. (2015). Moving object detection with a freely moving camera via background motion subtraction. IEEE Trans. Circuits Syst. Video Technol..

[33] Zhu Y., Elgammal A. A multilayer-based framework for online background subtraction with freely moving cameras. Proceedings of the 2017 IEEE International Conference on Computer Vision (ICCV).

[34] Trefethen L.N., Bau D. (1997). Numerical Linear Algebra.

[35] Hu J., Tang H. (2007). Numerical Method of Differential Equation.

[36] Perazzi F., Pont-Tuset J., McWilliams B., Van Gool L., Gross M., Sorkine-Hornung A. A benchmark dataset and evaluation methodology for video object segmentation. Proceedings of the 2016 IEEE Conference on Computer Vision and Pattern Recognition (CVPR).

[37] Sun D., Roth S., Black M. Secrets of optical flow estimation and their principles. Proceedings of the IEEE Conference on Computer Vision and Pattern Recognition, CVPR 2010.

